# Loneliness and Frailty Among Middle-Aged and Aging Sexual Minority Men Living With or Without HIV: A Longitudinal Cross-Lagged Panel Analysis

**DOI:** 10.1093/geroni/igad113

**Published:** 2023-10-21

**Authors:** Paula Meireles, Deanna Ware, Ana Henriques, Karen Nieves-Lugo, Valentina Stosor, Mark Brennan-Ing, Steven Meanley, Sabina Haberlen, Chukwuemeka N Okafor, Steve Shoptaw, M Reuel Friedman, Michael Plankey

**Affiliations:** EPIUnit–Instituto de Saúde Pública, Universidade do Porto, Rua das Taipas, Porto, Portugal; Laboratório para a Investigação Integrativa e Translacional em Saúde Populacional (ITR), Universidade do Porto, Rua das Taipas, Porto, Portugal; Division of General Internal Medicine, Department of Medicine, Georgetown University Medical Center, Washington, District of Columbia, USA; EPIUnit–Instituto de Saúde Pública, Universidade do Porto, Rua das Taipas, Porto, Portugal; Laboratório para a Investigação Integrativa e Translacional em Saúde Populacional (ITR), Universidade do Porto, Rua das Taipas, Porto, Portugal; Latino Health Research Center, Department of Psychology, The George Washington University, Washington, District of Columbia, USA; Divisions of Infectious Diseases and Organ Transplantation, Northwestern University Feinberg School of Medicine, Chicago, Illinois, USA; Brookdale Center for Healthy Aging at Hunter College, City University of New York, New York, New York, USA; Department of Family and Community Health, University of Pennsylvania School of Nursing, Philadelphia, Pennsylvania, USA; Department of Epidemiology, Johns Hopkins University Bloomberg School of Public Health, Baltimore, Maryland, USA; Department of Medicine, Division of Infectious Diseases, Long School of Medicine, University of Texas Health Science Center San Antonio, San Antonio, Texas, USA; Departments of Family Medicine and Psychiatry and Family Medicine, University of California Los Angeles, Los Angeles, California, USA; Department of Urban-Global Public Health, School of Public Health, Rutgers University, Piscataway, New Jersey, USA; Division of General Internal Medicine, Department of Medicine, Georgetown University Medical Center, Washington, District of Columbia, USA

**Keywords:** Aging, Frailty, Multicenter AIDS cohort study, Loneliness, Sexual minority men, United States

## Abstract

**Background and Objectives:**

Loneliness is associated with frailty among older adults (60+), and there is evidence suggesting that this association may be bidirectional. However, there is limited evidence of this relationship over time among middle-aged and aging sexual minority men. We explored the bidirectional relationship between loneliness and frailty over 2 years among sexual minority men living with or without human immunodeficiency virus (HIV) from the Healthy Aging substudy of the Multicenter AIDS Cohort Study.

**Research Design and Methods:**

We used data from 1 118 men (561 living with HIV; 557 living without HIV) aged 40 years or older with measurement of frailty or loneliness at Times 1 (September 2016 to March 2017) and 2 (September 2018 to March 2019). Descriptive statistics were generated. We used autoregressive cross-lagged panel analysis to examine the bidirectional association between frailty and loneliness at both time points while adjusting for time-stable and time-dependent covariates at Time 1. Adjusted odds ratios (aORs) and 95% confidence intervals (CIs) were generated.

**Results:**

The estimated prevalence of loneliness at both time points was 35.5%. The estimated prevalence of frailty at Times 1 and 2 were 7.8% and 12.1%, respectively. Participants reporting loneliness at Time 1 had greater odds of being frail at Time 2 (aOR = 2.14; 95% CI: 1.23–3.73). Frailty at Time 1 was not associated with loneliness at Time 2 (aOR = 1.00; 95% CI: .44–2.25). The autoregressive effects of frailty (aOR = 23.43; 95% CI: 11.94–46) and loneliness (aOR = 13.94; 95% CI: 9.42–20.61) were large.

**Discussion and Implications:**

Men who felt lonely had higher odds of being frail 2 years later while the reciprocal association was not shown. This suggests that loneliness preceded frailty and not the other way around. Early and frequent assessments of loneliness may present opportunities for interventions that minimize the risk of frailty among sexual minority men living with and without HIV.

**Translational Significance:** Loneliness is a risk factor for frailty, but some evidence suggests this association may be bidirectional. In this study among sexual minority men, aged 40 years or older, we found that those who felt lonely had higher odds of being frail 2 years later, while the reciprocal association was not shown. This suggests that loneliness precedes frailty but not the other way around. Additionally, the prevalence of loneliness and frailty remained stable over a 2-years’ of time. Promoting opportunities for interventions that reduce loneliness among aging sexual minority men can reduce frailty and other negative consequences of loneliness.

## Background and Objectives

Frailty is a state of high vulnerability to adverse outcomes, such as loss of independence, falls, institutionalization, and mortality (1,2). Fried et al. proposed a phenotype of frailty, which has been widely used, defined as the presence of 3 or more of the following 5 clinical measures: Unintentional weight loss, exhaustion, low physical activity, slowness, and weakness (3).

Since the advent of highly effective antiretroviral therapy in the mid-1990s, there has been extended survival of people living with human immunodeficiency virus (HIV). However, people living with face an earlier and greater burden of comorbidities compared with those living without HIV (4,5). Likewise, they are at higher risk of frailty than the general population and their HIV-negative counterparts (6–8). Nevertheless, in comparable populations of middle-aged (≥45 years old) HIV-positive and -negative individuals, HIV status did not modify the increased risk of mortality of frail individuals compared with nonfrail individuals (9). However, in another study, this risk was higher for older adults aged 65 years and older who felt lonely or socially isolated in addition to being frail (10).

Loneliness has been deemed a public health problem worldwide (11–14) and can be defined as the “feeling of isolation regardless of objective social network size” (15). It is, therefore, a subjective experience reflecting the discrepancy between one’s desired and one’s actual level of social relationships (16,17). In the context of HIV, loneliness is higher among older adults with HIV than among those without HIV (18,19) and among sexual minority individuals than among heterosexual individuals (20). Loneliness is associated with frailty among older adults in many countries and in both cross-sectional and longitudinal studies (21–25). The longitudinal studies investigating the relationship between loneliness and frailty assume loneliness as a risk factor for frailty but there is evidence suggesting that this association may be bidirectional (21,26).

To our knowledge, this bidirectional relationship between loneliness and frailty among sexual minority men, aged 40 years or older living with or without HIV, has not been explored yet. Therefore, we aimed to assess the bidirectional association between frailty and loneliness over 2 years among sexual minority men living with and without HIV enrolled in the Healthy Aging substudy of the Multicenter AIDS Cohort Study (MACS).

## Research Design and Methods

### Multicenter AIDS Cohort Study

Multicenter AIDS Cohort Study (MACS) is a prospective cohort study of sexual minority men living with and without HIV. From 1984 to 2019, 7 352 participants were enrolled across 4 sites in the United States: Baltimore, Maryland/Washington, DC; Chicago, Illinois; Los Angeles, California; and Pittsburgh, Pennsylvania/Columbus, Ohio. Participants attended semiannual clinic visits that used audio computer-assisted self-interview, which means that participants listened to prerecorded questions of a structured questionnaire through headphones and answered them on a computer, and a standardized clinical examination to collect demographic information, medical history, behavioral assessments, and biospecimens. Details on the MACS study design have been described elsewhere (27,28). Institutional review boards at each respective study site approved the MACS protocol and informed consent was obtained from all study participants.

### Understanding Patterns of Healthy Aging in Men Who Have Sex With Men

The Understanding Patterns of Healthy Aging Among Men Who Have Sex with Men substudy of the MACS seeks to understand the psychosocial resiliencies that promote healthy aging among middle-aged and older sexual minority men with and without HIV infection (29). The substudy was conducted over 6 MACS visits from April 2016 to March 2019. Eligible MACS participants for this substudy had to be at least 40 years old on April 2016, active in the study (attended a study visit within the 2 years prior to April 2016), and reported at least 1 incidence of sexual intercourse with another man since enrolling in the MACS. There was a total of 1 317 MACS participants enrolled in this substudy. For this work, we used information from 2 of the 6 visits of the substudy: Time 1 (September 2016 to March 2017) and Time 2 (September 2018 to March 2019). The current analyses included 1 118 participants (84.9%) with frailty or loneliness data at Times 1 and 2.

### Primary Measurements

Loneliness was assessed using the UCLA 3-Item Loneliness Scale (30) at Times 1 and 2. The questions were as follows: (1) How often do you feel that you lack companionship?; (2) How often do you feel left out?; and (3) How often do you feel isolated from others? The scale uses 3 response categories: “hardly ever” (scored 1), “some of the time” (scored 2), and “often” (scored 3). Responses were totaled, with values ranging from 3 to 9. Scores were then categorized into a dichotomous variable: “not lonely” (<6) and “lonely” (≥6) (31,32).

The definition for frailty at Times 1 and 2 was adopted within the MACS in 2008 using the Fried Frailty Phenotype (3,33,34). It was defined as the presence of 3 or more of the following clinical measures: (1) weakness (grip strength measured using a dynamometer less than the 20th percentile of HIV-negative participants); (2) slowness (timed walk of 4 m that is more than the 80th percentile of HIV-negative participants); (3) unintentional weight loss of at least 10 lb (an affirmative response, “yes,” to the question: “Since your last visit, have you had unintentional weight loss of at least 10 pounds?”); (4) reported exhaustion during work or other activities (an affirmative response, “yes,” to the question: “During the past 4 weeks, as a result of your physical health, have you had difficulty performing your work or other activities [for example, it took extra effort]?”); and (5) low physical activity due to health limitation (an affirmative response, “yes, limited a lot,” to the question: “Does your health now limit you in vigorous activities, such as running, lifting heavy objects, participating in strenuous sports?”). Not meeting at least 3 of the aforementioned criteria was categorized as nonfrail.

### Covariates

All covariates were assessed at Time 1. Participants’ chronological age was calculated from the self-reported date of birth and date of visit. Race and ethnicity were categorized as Hispanic, non-Hispanic Black, non-Hispanic White, and Other (Multi-race, Asian/Pacific Islander, and Native American). Education was categorized as less than a high school diploma, high school diploma, at least some college, and at least some graduate school. Age discrepancy was calculated as the difference between subjective age (“What age [years] do you feel most of the time?”) and chronological age. Age discrepancy was categorized into 3 categories: older subjective age (subjective age > chronological age); no age discrepancy (subjective age = chronological age); and younger subjective age (subjective age < chronological age) (35). HIV status (participants living with HIV/participants living without HIV) was assessed using an enzyme-linked immunosorbent assay with a confirmatory western blot on all MACS participants. Participants living with HIV included all participants with a confirmed positive western blot at their baseline MACS visit and those who seroconverted at any time during follow-up in the MACS. Depressive symptoms were defined using the Center for Epidemiological Studies—Depression scale, with scores greater than or equal to 16 indicating the presence of depressive symptoms (36). The presence of the following comorbidities were assessed: (1) high blood pressure (systolic blood pressure ≥140 mm Hg or diastolic blood pressure ≥90 mm Hg or diagnosed with hypertension and use of medication); (2) diabetes (high fasting blood glucose (≥126 mg/dL) or elevated HbA1c (≥6.5) or previous clinic diagnosis with the use of medication); (3) liver disease (serum glutamic pyruvic transaminase or serum glutamic oxaloacetic transaminase >150 U/L); (4) kidney disease (estimated glomerular filtration rate <60 mL/min/1.73 m^2^ body surface area using the Modification of Diet in Renal Disease equation (37) or urine protein-to-creatinine ratio ≥200 mg protein/1 g creatinine); (5) dyslipidemia (fasting total cholesterol ≥200 mg/dL, low-density lipoprotein cholesterol ≥130 mg/dL, high-density lipoprotein cholesterol <40 mg/dL, or triglycerides ≥150 mg/dL, or use of lipid-lowering medications with self-report of a previous clinical diagnosis); and (6) current hepatitis C infection defined as detectable hepatitis C RNA in serum; based on previous work from Althoff et al. and Nieves-Lugo et al. (33–35) and using the updated MACS definitions. The comorbidities were summed and reported as a count from 0 to 6.

### Statistical Methods

**Figure 1.**
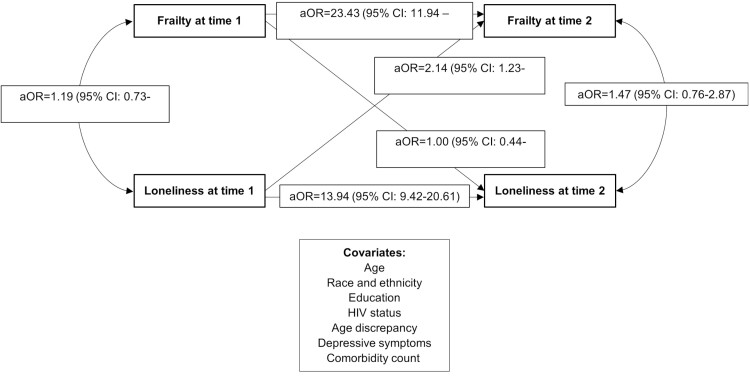
Cross-lagged panel analysis diagram. aOR = adjusted odds ratio.

We generated descriptive statistics of the primary measures (loneliness and frailty) and the covariates, overall and by HIV status, using absolute and relative frequencies and medians and 25th and 75th percentiles (P25–P75) where appropriate. We used a cross-lagged panel analysis to examine the reciprocal relationship between frailty and loneliness at Times 1 and 2 with adjustments for covariates measured at Time 1 (Figure 1) (38). The model generated estimates for cross-lagged and autoregressive effects. Cross-lagged effects refer to the association of loneliness with the future occurrence of frailty and vice versa. The inclusion of the autoregressive effect allowed for adjustment of the previous levels of loneliness and frailty as well as described the stability from Time 1 to Time 2 (38). We initially stratified analyses by HIV status to assess differences by HIV; however, because differences compared with the unstratified analyses were marginal, we retained HIV status as a covariate in the final models. In regard to missing data, the cross-panel analysis used full information maximum likelihood and used all data available when estimating models (39). We included the results of complete cases only in Supplementary Tables 1 and 2, but no substantial differences were found. We reported adjusted odds ratios (aORs) and 95% confidence intervals (CIs). Analyses were performed in SAS version 9.4 (Statistical Analysis Software Inc) and Mplus (Muthén & Muthén) (40).

## Results

Participants’ characteristics are shown in Table 1. The median age was 60 years (P25–P75: 54–66). Most participants were non-Hispanic White (68.8%), reported at least some college (85.6%), had a younger subjective age (81.8%), and had a median of 2 comorbid conditions (P25–P75: 1–3). Nearly, a quarter of the participants reported depressive symptoms (23.4%). The estimated prevalence of loneliness at both time points was 35.5%. The estimated prevalence of frailty at Times 1 and 2 were 7.8% and 12.1%, respectively.

**Table 1.** Participants’ Characteristics by HIV Status

Primary measures and covariates	Participants living without HIV (*n* = 561)	Participants living with HIV (*n* = 557)	Overall (*n* = 1 118)
Age, median (P25–P75), y	62 (56–68)	57 (52–63)	60 (54–66)
Race and ethnicity, *n* (%)			
Hispanic	28 (5.0)	66 (11.9)	94 (8.4)
Non-Hispanic Black	69 (12.3)	162 (29.1)	231 (20.7)
Non-Hispanic White	452 (80.6)	317 (56.9)	769 (68.8)
Other	12 (2.1)	12 (2.2)	24 (2.1)
Education, *n* (%)			
Less than High School	10 (1.8)	19 (3.4)	29 (2.6)
High school	42 (7.5)	70 (12.6)	112 (10.0)
College	232 (41.4)	272 (48.8)	504 (45.1)
Graduate school	268 (47.8)	185 (33.2)	453 (40.5)
Missing	9 (1.6)	11 (2.0)	20 (1.8)
Age discrepancy, *n* (%)			
Younger than subjective age	471 (84.0)	443 (79.5)	914 (81.8)
No age discrepancy	60 (10.7)	50 (9.0)	110 (9.8)
Older than subjective age	28 (5.0)	59 (10.6)	87 (7.8)
Missing	2 (0.4)	5 (0.9)	7 (0.6)
Depressive symptoms, *n* (%)			
Depressive symptoms	209 (19.2)	300 (27.6)	509 (23.4)
No depressive symptoms	847 (78.0)	761 (70.0)	1608 (74.0)
Missing	30 (2.8)	26 (2.4)	56 (2.6)
Comorbidities count, median (P25–P75)	2 (1–2)	2 (1–3)	2 (1–3)
Loneliness, at Time 1, *n* (%)^*^			
No loneliness	292 (68.4)	237 (60.3)	529 (64.5)
Loneliness	135 (31.6)	156 (39.7)	291 (35.5)
Loneliness, at Time 2 *n* (%)^*^			
No loneliness	278 (65.7)	246 (63.1)	524 (64.5)
Loneliness	145 (34.3)	144 (36.9)	289 (35.5)
Frailty at Time 1, *n* (%)^*^			
No frailty	395 (93.8)	357 (90.4)	752 (92.2)
Frailty	26 (6.2)	38 (9.6)	64 (7.8)
Frailty at Time 2, *n* (%)^*^			
No frailty	367 (88.4)	333 (87.4)	700 (87.9)
Frailty	48 (11.6)	48 (12.6)	96 (12.1)

*Notes*: HIV = human immunodeficiency virus; P25–P75 = 25th–75th percentiles.

^*^Prevalence estimated from cross-panel analysis.

Among participants living with HIV, the median age was 57 years (IQR: 52–63), 56.9% were non-Hispanic White, 82.0% reported at least some college, 79.5% felt younger than their chronological age, and had a median comorbidity count of 2 (P25–P75: 1–3). Depressive symptoms were reported by 27.6% of participants living with HIV. The estimated prevalence of loneliness at Times 1 and 2 were 39.7% and 36.9%, respectively. The estimated prevalence of frailty at Times 1 and 2 were 9.6% and 12.6%, respectively.

Among participants living without HIV, the median age was 62 years (IQR: 56–68), 80.6% were non-Hispanic White, 89.2% reported at least some college, 84.0% felt younger than their chronological age, and had a median comorbidity count of 2 (IQR: 1–2). Depressive symptoms were reported by 19.2% of participants living with HIV. The estimated prevalence of loneliness at Times 1 and 2 were 31.6% and 34.3%, respectively. The estimated prevalence of frailty at Times 1 and 2 were 6.2% and 11.6%, respectively.

### Longitudinal Cross-Lagged Association Between Loneliness and Frailty

After adjusting for covariates, participants reported feeling lonely at Time 1 had greater odds of being frail at Time 2 (aOR = 2.14 [95% CI: 1.23–3.73]). Frailty at Time 1 did not have a statistically significant association with loneliness at Time 2 (aOR = 1.00 [95% CI: 0.44–2.25]; Table 2). The autoregressive effects of frailty (aOR = 23.43 [95% CI: 11.94–46.00]) and loneliness (aOR = 13.94 [95% CI: 9.42–20.61]) were large, indicating stability in responses across the 2 time points.

**Table 2.** Reciprocal Association Between Loneliness and Frailty, Adjusting for Covariates

Primary measures and covariates	Odds ratio (95% CI)			
	Loneliness at Time 1	Frailty at Time 1	Loneliness at Time 2	Frailty at Time 2
Loneliness at Time 1 (vs no loneliness)	—	1.19 (0.73–1.96)	13.94 (9.42–20.61)	2.14 (1.23–3.73)
Loneliness at Time 2 (vs no loneliness)	—	—	—	1.47 (0.76–2.87)
Frailty at Time 1 (vs no frailty)	1.19 (0.73–1.96)	—	1.00 (0.44–2.25)	23.43 (11.94–46.00)
Frailty at Time 2 (vs no frailty)	—	—	1.47 (0.76–2.87)	—
Age ≥ 60 y (vs <60 y)	1.33 (0.97–1.83)	3.34 (1.96–5.68)	0.74 (0.49–1.12)	2.23 (1.22–4.08)
Race and ethnicity				
Black (vs White)	0.94 (0.64–1.38)	1.32 (0.74–2.34)	1.28 (0.77–2.12)	2.29 (1.21–4.32)
Hispanic (vs White)	0.83 (0.48–1.44)	1.79 (0.82–3.88)	0.83 (0.42–1.66)	1.53 (0.57–4.08)
Other (vs White)	0.66 (0.24–1.82)	1.48 (0.39–5.59)	0.19 (0.04–0.89)	0.43 (0.05–4.12)
Education				
Less than high school (vs high school)	1.21 (0.43–3.36)	1.61 (0.51–5.07)	1.89 (0.52–6.92)	0.45 (0.08–2.46)
At least some college (vs high school)	0.99 (0.60–1.63)	0.82 (0.42–1.62)	1.20 (0.61–2.35)	0.62 (0.28–1.37)
Graduate school (vs high school)	0.91 (0.54–1.54)	0.58 (0.28–1.22)	1.13 (0.56–2.25)	0.98 (0.43–2.23)
Participants living with HIV (vs Participants living without HIV)	1.22 (0.91–1.64)	1.11 (0.70–1.75)	0.63 (0.42–0.94)	0.66 (0.38–1.13)
Age discrepancy				
Younger (vs no age discrepancy)	0.70 (0.44–1.12)	0.40 (0.22–0.76)	0.59 (0.32–1.11)	1.05 (0.42–2.57)
Older (vs no age discrepancy)	2.05 (1.02–4.11)	1.55 (0.70–3.43)	1.85 (0.73–4.65)	2.70 (0.88–8.31)
Depressive symptoms (vs no depressive symptoms)	6.96 (4.85–10.00)	2.57 (1.56–4.25)	1.88 (1.16–3.05)	0.93 (0.49–1.78)
Comorbidities				
Each increase in count of comorbidities	1.08 (0.94–1.24)	1.27 (1.04–1.55)	1.17 (0.97–1.40)	1.45 (1.14–1.85)

*Note*: 95% CI = confidence interval; HIV = human immunodeficiency virus.

### Associations Between Loneliness, Frailty, and Covariates at Time 1

Feeling older than one’s chronological age (aOR = 2.05 [95% CI: 1.02–4.11]) and reporting depressive symptoms (aOR = 6.96 [95% CI: 4.85–10.00]) were associated with increased odds of loneliness. Being 60 years or older (aOR = 3.34 [95% CI: 1.96–5.68]), reporting depressive symptoms (aOR = 2.57 [95% CI: 1.56–4.25]), and having an increased number of comorbid conditions (aOR = 1.27 [95% CI: 1.04–1.55]) were associated with increased odds of frailty. The associations between HIV status and loneliness (aOR = 1.22 [95% CI: 0.91–1.64]) or frailty (aOR = 1.11 [95% CI: 0.70–1.75]) were not statistically significant, but the first association was more compatible with a positive association (Table 2).

### Associations Between Loneliness, Frailty, and Covariates at Time 2

Living with HIV (aOR = 0.63 [95% CI: 0.42–0.94]) was associated with decreased odds of loneliness, while reporting depressive symptoms (aOR = 1.88 [95% CI: 1.16–3.05]) was associated with increased odds of loneliness. Older age (≥60 years old; aOR = 2.23 [95% CI: 1.22–4.08]), an increased number of comorbid conditions (aOR = 1.45 [95% CI: 1.14–1.85]), and identifying as non-Hispanic Black (aOR = 2.29 [95% CI: 1.21–4.32]) were positively associated with frailty. The association between HIV status and frailty was not statistically significant (aOR = 0.66 [95% CI: 0.38–1.13]) but was compatible with a decrease in odds (Table 2).

## Discussion and Implications

These results showed that feeling lonely increased the risk of being frail 2 years later, independently of previously presenting frailty, while frailty did not predict future loneliness. The predictive ability of loneliness with future frailty had been previously shown in male and female adults aged 50 years or older (21,24,25,41). However, the predictive ability of frailty in relation to loneliness was not shown, unlike what previous studies among older adults (60+) suggested (21,26). It is important to note that our participants included also men aged 40 or older and the literature on the association between frailty and loneliness in middle-aged adults younger than 50 is unknown. We also found that loneliness and frailty remained stable across the 2 visits as shown by the large autoregressive effects estimated for both constructs. We found no cross-sectional association between loneliness and frailty at Time 1 and Time 2.

The high stability of both constructs over time may partially explain the lack of association of frailty with future loneliness because the progression to loneliness can be mostly explained by a high prevalence of loneliness in Time 1, which already affected more than one-third of the sample. We can speculate that loneliness could be already present in this cohort of men and that between the 2-time points considered (September 2016 to March 2017 and September 2018 to March 2019), there was not enough time to detect any effect of previous frailty in loneliness. A loneliness prevalence of 35.5% in the study sample is similar to a previous estimate for the general U.S. population aged 45 years and older in 2018 of 35%, but lower than that found among lesbian, gay, bisexual, transgender, and questioning (or queer; 49%) persons in the same study (42).

Despite the higher prevalence of loneliness compared with the general population, gay and bisexual men seem to follow a similar loneliness trajectory like other adults in which it gradually diminishes through the middle adult years (i.e., 50+) and then increases in old age (i.e., 65+) (43,44). In our sample, the median age and 75th percentile were 60 and 66 years old, respectively, which means that we are likely capturing a time in life when loneliness is at its lowest throughout middle and older age before starting an increasing trajectory at around 65–70 years of age (43). Moreover, feelings of loneliness may differ in terms of temporal persistence, being either chronic or short-lived (17). Thus, the chronicity of loneliness and its longitudinal measurement is an important dimension to consider in future studies because it differs from short-lived loneliness in terms of etiology as well as treatment implications (45). With all these considerations in mind, longer follow-up periods are needed to assess whether frailty can predict loneliness in this population.

In its turn, the predictive role of loneliness in relation to frailty—and for components of frailty such as gait speed, fatigue, and physical inactivity, and for activities of daily living or cognitive impairment—had been previously shown among older adults (50+) (22,46). Additionally, greater loneliness was also shown to be associated with lower odds of reversion from a prefrail or frail state to a robust (nonfrail) (24). These findings showed that tackling loneliness could not only decrease the risk of progressing to frail from a nonfrail state, but also increase the odds of reversing to a robust state in the future (24). A recent study in the same population as our study found that those reporting a higher level of social environmental resiliencies measured as perceived social support, social bonding, and psychological sense of community were less likely to experience loneliness than those who did not (31). Social environmental resiliencies were framed as overtime buffers against symptoms of loneliness in a context of stigma and discrimination faced by many MSM throughout life (31). Promoting opportunities to increase, or maintain, high support and social bonding from social networks, and a strong attachment to the community with special investment among those socially disconnected, has the potential to reduce loneliness (31) and, by that, also reduce future frailty and other deleterious effects of loneliness.

In accordance with previous cross-sectional and longitudinal studies, we also found that an increasing count of comorbidities at Time 1 was associated with loneliness and frailty at the 2 time points (47). Furthermore, depressive symptoms were very strongly associated with loneliness and frailty at Time 1, while a longitudinal weaker association was only found for loneliness. These associations had been previously described; however, the lack of association between depressive symptoms and frailty at Time 2 is unlike in previous studies (48–50).

Also noteworthy was the positive cross-sectional association of feeling older than chronological age with only loneliness at Time 1, while being aged 60 years or older was only strongly positively associated with frailty at Time 1 and Time 2 and negatively with loneliness at Time 2 (even if not reaching statistical significance). Subjective age is an independent predictor of several age-related outcomes and, while most individuals feel younger than their chronological age, those who feel older usually have poorer health, behavioral, and cognitive outcomes, such as emotional well-being and activities of daily living (51). Therefore, the association of an older subjective age with feeling lonely found in the current study is not surprising. In addition, the lack of association between chronological age and loneliness reinforces that this small subgroup that feels older than chronological age may be even more subject to the deleterious effects of negative age stereotypes as they are also more strongly influenced by ageist stereotypes (52). For instance, internalized ageism, a form of ingroup discrimination in which older adults marginalize and discriminate against other older people, likely as a result of a lifespan of internalizing negative age stereotypes, has a wide range of negative health impacts 53–55. Although marginal in our study, this finding could potentially serve as a preventive measure because it may be possible to intervene in an individual’s perception of their subjective age, whereas chronological age is nonmodifiable.

Unexpectedly, we found that both older participants and participants living with HIV were less likely to report loneliness at Time 2, while a positive, not statistically significant, effect was shown at Time 1. There is a possible explanation for this. Because the autoregressive effect of loneliness at Time 1 was included in the model, we measured the odds of older participants and participants living with HIV feeling lonely at Time 2 independently of previous levels of loneliness. In the case of HIV status, even though participants living with HIV reported feeling lonely more frequently than those not living with HIV at both time points, this proportion diminished from 39.7% in Time 1 to 36.9% in Time 2 among participants living with HIV, reducing the difference from 8% to 2% points between participants living with HIV and participants living without HIV (Table 1). This suggests that participants living with HIV were less likely to become lonely from Time 1 to Time 2 than participants living without HIV.

Our findings were generated using an autoregressive cross-lagged analysis that allowed us to estimate the reciprocal association of frailty and loneliness independently of the previous level of each of these constructs. This approach had not yet been used on this topic, to our knowledge, and allowed us to minimize bias in the estimation of reciprocal associations (38,53,54). Another strength of this study was the use of the Fried frailty phenotype and the UCLA Loneliness Scale, which are widely used and allow comparison with other studies.

This study did have limitations. The findings have limited generalizability given the convenience sampling for the recruitment design, and its limited diversity by consisting mostly of non-Hispanic White sexual minority men (28). We also had a considerable (15.1%) proportion of missing values for the primary measures in Time 2. However, the sensitivity analysis using only those with information for loneliness and frailty at both time points showed similar results (Supplementary Tables 1 and 2). Another limitation was the short time frame between the 2 visits, which may not have been enough to show the expected reciprocal association between frailty and loneliness. A longer follow-up time would be required to define with higher confidence whether loneliness and frailty have a reciprocal association or whether loneliness precedes frailty as our results suggest. Additionally, following up with these men to an older age would be important to capture the full middle-age to older age trajectory of loneliness.

In conclusion, middle-aged and older sexual minority men living with or without HIV who feel lonely have higher odds of being frail at a later time in life, while the reciprocal association was not shown. This suggests that loneliness precedes frailty and not the other way around. Additionally, both loneliness and frailty remained stable over time. Early assessment and mitigation of loneliness and frailty among these men are essential to healthy wellness in aging.

## Supplementary Material

igad113_suppl_Supplementary_Tables_1-2

## References

[1] Fried LP , Ferrucci L, Darer J, Williamson JD, Anderson G. Untangling the concepts of disability, frailty, and comorbidity: implications for improved targeting and care. J Gerontol A Biol Sci Med Sci. 2004;59(3):M255–M263. 10.1093/gerona/59.3.m255 15031310

[2] Ahmed N , Mandel R, Fain MJ. Frailty: An emerging geriatric syndrome. Am J Med. 2007;120(9):748–753. 10.1016/j.amjmed.2006.10.018 17765039

[3] Fried LP , Tangen CM, Walston J, et al. Cardiovascular Health Study Collaborative Research Group. Frailty in older adults: Evidence for a phenotype. J Gerontol A Biol Sci Med Sci. 2001;56(3):M146–M156. 10.1093/gerona/56.3.m146 11253156

[4] Althoff KN , Smit M, Reiss P, Justice AC. HIV and ageing: Improving quantity and quality of life. Curr Opin HIV AIDS. 2016;11(5):527–536. 10.1097/COH.0000000000000305 27367780 PMC5084838

[5] Van Epps P , Kalayjian RC. Human immunodeficiency virus and aging in the era of effective antiretroviral therapy. Infect Dis Clin North Am. 2017;31(4):791–810. 10.1016/j.idc.2017.07.007 28916384

[6] Bloch M. Frailty in people living with HIV. AIDS Res Ther. 2018;15(1):19. 10.1186/s12981-018-0210-2 30445966 PMC6240180

[7] Kooij KW , Wit FWNM, Schouten J, et al. AGEhIV Cohort Study Group. HIV infection is independently associated with frailty in middle-aged HIV type 1-infected individuals compared with similar but uninfected controls. AIDS. 2016;30(2):241–250. 10.1097/QAD.0000000000000910 26684821

[8] Verheij E , Wit FW, Verboeket SO, et al. Frequency, risk factors, and mediators of frailty transitions during long-term follow-up among people with HIV and HIV-Negative AGEhIV Cohort participants. J Acquir Immune Defic Syndr (1999). 2021;86(1):110–118. 10.1097/QAI.0000000000002532 PMC7722459 33105395

[9] Verheij E , Kirk GD, Wit FW, et al. AGEhIV Cohort. Frailty is associated with mortality and incident comorbidity among middle-aged human immunodeficiency virus (HIV)–Positive and HIV-Negative participants. J Infect Dis. 2020;222(6):919–928. 10.1093/infdis/jiaa010 31956893 PMC7430168

[10] Hoogendijk EO , Smit AP, van Dam C, et al. Frailty combined with loneliness or social isolation: An elevated risk for mortality in later life. J Am Geriatr Soc. 2020;68(11):2587–2593. 10.1111/jgs.16716 32700319 PMC7689758

[11] Cacioppo JT , Cacioppo S. The growing problem of loneliness. Lancet. 2018;391(10119):426. 10.1016/S0140-6736(18)30142-9 PMC6530780 29407030

[12] Cotterell N , Buffel T, Phillipson C. Preventing social isolation in older people. Maturitas. 2018;113:80–84. 10.1016/j.maturitas.2018.04.014 29903652

[13] Gerst-Emerson K , Jayawardhana J. Loneliness as a public health issue: The impact of loneliness on health care utilization among older adults. Am J Public Health. 2015;105(5):1013–1019. 10.2105/AJPH.2014.302427 25790413 PMC4386514

[14] Surkalim DL , Luo M, Eres R, et al. The prevalence of loneliness across 113 countries: Systematic review and meta-analysis. BMJ Open. 2022;376:e067068. 10.1136/bmj-2021-067068 PMC8826180 35140066

[15] Freedman A , Nicolle J. Social isolation and loneliness: the new geriatric giants: Approach for primary care. Can Fam Physician. 2020;66(3):176–182. 32165464 PMC8302356

[16] Lim MH , Eres R, Vasan S. Understanding loneliness in the twenty-first century: an update on correlates, risk factors, and potential solutions. Soc Psychiatry Psychiatr Epidemiol. 2020;55(7):793–810. 10.1007/s00127-020-01889-7 32524169

[17] Peplau LA , Perlman D. Perspectives on loneliness. In: Loneliness: A Sourcebook of Current Theory, Research and Therapy. L. A. Peplau & D. Perlman (Eds.); 1982.

[18] Karpiak S , Shippy A, Cantor M. Research on Older Adults with HIV. 2006. 10.13140/RG.2.1.3656.4328

[19] Leyva-Moral JM , Martínez-Batlle F, Vázquez-Naveira M, Hernández-Fernández J, Villar-Salgueiro M. The experience of growing old while living with HIV in Spain: A phenomenological study. J Assoc Nurses AIDS Care. 2019;30(1):111–118. 10.1097/JNC.0000000000000032 30586088

[20] Gorczynski PP , Fasoli PF. Loneliness in sexual minority and heterosexual individuals: A comparative meta-analysis. J Gay Lesbian Ment Health. 2021;0(0):1–18. 10.1080/19359705.2021.1957742

[21] Gale CR , Westbury L, Cooper C. Social isolation and loneliness as risk factors for the progression of frailty: The English Longitudinal Study of Ageing. Age Ageing. 2018;47(3):392–397. 10.1093/ageing/afx188 29309502 PMC5920346

[22] Giné-Garriga M , Jerez-Roig J, Coll-Planas L, et al. Is loneliness a predictor of the modern geriatric giants? Analysis from the survey of health, ageing, and retirement in Europe. Maturitas. 2021;144:93–101. 10.1016/j.maturitas.2020.11.010 33358215

[23] Herrera-Badilla A , Navarrete-Reyes AP, Amieva H, Avila-Funes JA. Loneliness Is associated with frailty in community-dwelling elderly adults. J Am Geriatr Soc. 2015;63(3):607–609. 10.1111/jgs.13308 25800917

[24] Jarach CM , Tettamanti M, Nobili A, D’avanzo B. Social isolation and loneliness as related to progression and reversion of frailty in the Survey of Health Aging Retirement in Europe (SHARE). Age Ageing. 2021;50(1):258–262. 10.1093/ageing/afaa168 32915990 PMC7793602

[25] Sha S , Xu Y, Chen L. Loneliness as a risk factor for frailty transition among older Chinese people. BMC Geriatr. 2020;20(1):300. 10.1186/s12877-020-01714-5 32831020 PMC7446170

[26] Hoogendijk EO , Suanet B, Dent E, Deeg DJH, Aartsen MJ. Adverse effects of frailty on social functioning in older adults: Results from the Longitudinal Aging Study Amsterdam. Maturitas. 2016;83:45–50. 10.1016/j.maturitas.2015.09.002 26428078

[27] Farinpour R , Miller EN, Satz P, et al. Psychosocial risk factors of HIV morbidity and mortality: Findings from the Multicenter AIDS Cohort Study (MACS). J Clin Exp Neuropsychol. 2003;25(5):654–670. 10.1076/jcen.25.5.654.14577 12815503

[28] Kaslow RA , Ostrow DG, Detels R, Phair JP, Polk BF, Rinaldo CR. The multicenter AIDS Cohort Study: Rationale, organization, and selected characteristics of the participants. Am J Epidemiol. 1987;126(2):310–318. 10.1093/aje/126.2.310 3300281

[29] Egan JE , Haberlen SA, Meansley S, et al. Understanding patterns of healthy aging among men who have sex with men: Protocol for an Observational Cohort Study. JMIR Res Protoc. 2021;10(9):e25750. 10.2196/25750 34554100 PMC8498890

[30] Hughes ME , Waite LJ, Hawkley LC, Cacioppo JT. A Short scale for measuring loneliness in large surveys: results from two population-based studies. Res Aging. 2004;26(6):655–672. 10.1177/0164027504268574 18504506 PMC2394670

[31] De Jesus M , Ware D, Brown AL, et al. Social-environmental resiliencies protect against loneliness among HIV-Positive and HIV- negative older men who have sex with men: Results from the Multicenter AIDS Cohort Study (MACS). Soc Sci Med. 2021;272:113711. 10.1016/j.socscimed.2021.113711 33550066 PMC8900533

[32] Steptoe A , Shankar A, Demakakos P, Wardle J. Social isolation, loneliness, and all-cause mortality in older men and women. Proc Natl Acad Sci U S A. 2013;110(15):5797–5801. 10.1073/pnas.1219686110 23530191 PMC3625264

[33] Althoff KN , Jacobson LP, Cranston RD, et al. Multicenter AIDS Cohort Study (MACS). Age, comorbidities, and AIDS predict a frailty phenotype in men who have sex with men. J Gerontol A Biol Sci Med Sci. 2014;69(2):189–198. 10.1093/gerona/glt148 24127428 PMC4038242

[34] Nieves-Lugo K , Ware D, Althoff K, et al. Negative perception of aging is associated with frailty transitions within a cohort of sexual minority men. Innov Aging. 2021;5(4):igab035. 10.1093/geroni/igab035 34805554 PMC8599189

[35] Nieves-Lugo K , Ware D, Friedman MR, et al. Self-perception of aging among HIV-positive and HIV-negative participants in the Multicenter AIDS Cohort Study. AIDS Care. 2020;32(7):818–828. 10.1080/09540121.2019.1668536 31547674 PMC7085960

[36] Radloff LS. The CES-D scale: A self-report depression scale for research in the general population. Appl Psychol Measurement. 1977;1(3):385–401. 10.1177/014662167700100306

[37] Levey AS , Bosch JP, Lewis JB, Greene T, Rogers N, Roth D. A more accurate method to estimate glomerular filtration rate from serum creatinine: A new prediction equation Modification of Diet in Renal Disease Study Group. Ann Intern Med. 1999;130(6):461–470. 10.7326/0003-4819-130-6-199903160-00002 10075613

[38] Selig JP , Little TD. Autoregressive and cross-lagged panel analysis for longitudinal data. In B Laursen, TD Little, NA Card (Eds.), Handbook of developmental research methods. The Guilford Press. 2012;265–278.

[39] Enders CK , Bandalos DL. The relative performance of full information maximum likelihood estimation for missing data in structural equation models. Struct Equ Model Multidiscip J. 2001;8(3):430–457. 10.1207/S15328007SEM0803_5

[40] Muthén B , Muthén L. Mplus. In: Handbook of Item Response Theory. Chapman and Hall/CRC; 2017.

[41] Davies K , Maharani A, Chandola T, Todd C, Pendleton N. The longitudinal relationship between loneliness, social isolation, and frailty in older adults in England: A prospective analysis. Lancet Healthy Longev. 2021;2(2):e70–e77. 10.1016/S2666-7568(20)30038-6 36098160

[42] Thayer C , Anderson GO. Loneliness and Social Connections: A National Survey of Adults 45 and Older. AARP Research; 2018. 10.26419/res.00246.001

[43] Lam J , Campbell A. Trajectories of loneliness among older women and men: Variation by sexual identity? Gerontologist. 2022;63:328–337. 10.1093/geront/gnac058 PMC9960021 35452512

[44] Pinquart M , Sorensen S. Influences on loneliness in older adults: A meta-analysis. Basic Appl Soc Psychol. 2001;23(4):245–266. 10.1207/s15324834basp2304_2

[45] Heinrich LM , Gullone E. The clinical significance of loneliness: a literature review. Clin Psychol Rev. 2006;26(6):695–718. 10.1016/j.cpr.2006.04.002 16952717

[46] Shankar A , McMunn A, Demakakos P, Hamer M, Steptoe A. Social isolation and loneliness: prospective associations with functional status in older adults. Health Psychol. 2017;36(2):179–187. 10.1037/hea0000437 27786518

[47] Hajek A , Kretzler B, König HH. Multimorbidity, loneliness, and social isolation a systematic review. Int J Environ Res Public Health. 2020;17(22):8688. 10.3390/ijerph17228688 33238506 PMC7700324

[48] Soysal P , Veronese N, Thompson T, et al. Relationship between depression and frailty in older adults: A systematic review and meta-analysis. Ageing Res Rev. 2017;36:78–87. 10.1016/j.arr.2017.03.005 28366616

[49] Borges MK , Romanini CV, Lima NA, et al. Longitudinal association between late-life depression (LLD) and frailty: Findings from a Prospective Cohort Study (MiMiCS-FRAIL). J Nutr Health Aging. 2021;25(7):895–902. 10.1007/s12603-021-1639-x 34409968 PMC8103429

[50] Aprahamian I , Suemoto CK, Lin SM, et al. Depression is associated with self-rated frailty in older adults from an outpatient clinic: A prospective study. Int Psychogeriatr. 2019;31(3):425–434. 10.1017/S104161021800100X 30099972

[51] Ryff CD , Krueger RF. The Oxford Handbook of Integrative Health Science. Oxford University Press; 2018.

[52] Eibach RP , Mock SE, Courtney EA. Having a “senior moment”: induced aging phenomenology, subjective age, and susceptibility to ageist stereotypes. J Exp Soc Psychol. 2010;46(4):643–649. 10.1016/j.jesp.2010.03.002

[53] Cole DA , Maxwell SE. Testing mediational models with longitudinal data: Questions and tips in the use of structural equation modeling. J Abnorm Psychol. 2003;112(4):558–577. 10.1037/0021-843X.112.4.558 14674869

[54] Gollob HF , Reichardt CS. Taking account of time lags in causal models. Child Dev. 1987;58(1):80–92. 3816351

