# Supplementary material for: Loneliness and Frailty Among Middle-Aged and Aging Sexual Minority Men Living With or Without HIV: A Longitudinal Cross-Lagged Panel Analysis
Source: Innov Aging. 2023 Oct 21;7(9):igad113. doi: 10.1093/geroni/igad113 (PMC10652703; doi:10.1093/geroni/igad113)
Supplement: igad113_suppl_Supplementary_Tables_1-2 [file igad113_suppl_supplementary_tables_1-2.docx]

**Online Supplementary Material**

Supplemental Table 1. Complete Case Description of Population Characteristics by HIV Status (N=787)

| **Primary Measures and Covariates** | **Participants living without HIV** | **Participants living with HIV** | **Overall** |
| --- | --- | --- | --- |
| **N** | 406 | 381 | 787 |
| **Age, median (P25-P75), y** | 62 (56-68) | 57 (52-63) | 60 (54-66) |
| **Race and ethnicity, n (%)** |  |  |  |
| Hispanic | 21 (5.2) | 52 (13.7) | 73 (9.3) |
| Non-Hispanic Black | 42 (10.3) | 109 (28.6) | 151 (19.2) |
| Non-Hispanic White | 335 (82.5) | 210 (55.1) | 545 (69.3) |
| Other | 8 (2.0) | 10 (2.6) | 18 (2.3) |
| **Education, n (%)** |  |  |  |
| Less than high school | 5 (1.2) | 14 (3.7) | 19 (2.4) |
| High school | 29 (7.1) | 44 (11.6) | 73 (9.3) |
| College | 168 (41.4) | 183 (48.0) | 351 (44.6) |
| Graduate school | 203 (50.0) | 139 (36.5) | 342 (43.5) |
| Missing | 1 (0.3) | 1 (0.3) | 2 (0.3) |
| **Age discrepancy, n (%)** |  |  |  |
| Younger than subjective age | 355 (87.4) | 306 (80.3) | 661 (84.0) |
| No age discrepancy | 34 (8.4) | 31 (8.1) | 65 (8.3) |
| Older than subjective age | 16 (3.9) | 43 (11.3) | 59 (7.5) |
| Missing | 1 (0.3) | 1 (0.3) | 2 (0.3) |
| **Depressive symptoms, n (%)** |  |  |  |
| Depressive Symptoms | 197 (19.8) | 261 (26.9) | 458 (23.3) |
| No depressive symptoms | 792 (79.5) | 701 (72.3) | 1493 (76.0) |
| Missing | 7 (0.7) | 7 (0.7) | 14 (0.7) |
| **Comorbidities count, median (P25-P75)** | 2 (1-2) | 2 (1-3) | 2 (1-3) |
| **Loneliness at time 1, n (%)** |  |  |  |
| No loneliness | 286 (70.4) | 229 (60.1) | 515 (65.4) |
| Loneliness | 120 (29.6) | 152 (39.9) | 272 (34.6) |
| **Loneliness at time 2, n (%)** |  |  |  |
| No loneliness | 275 (67.7) | 239 (62.7) | 514 (65.3) |
| Loneliness | 131 (32.3) | 142 (37.3) | 273 (34.7) |
| **Frailty at time 1, n (%)** |  |  |  |
| No frailty | 382 (94.1) | 347 (91.1) | 729 (92.6) |
| Frailty | 24 (5.9) | 34 (8.9) | 58 (7.4) |
| **Frailty at time 2, n (%)** |  |  |  |
| No frailty | 360 (88.7) | 333 (87.4) | 693 (88.1) |
| Frailty | 46 (11.3) | 48 (12.6) | 94 (11.9) |

Abbreviations: P25-P75, 25th-75th percentiles.

Supplemental Table 2. Complete Case Analysis of the Reciprocal Relationship Between Loneliness and Frailty, Adjusting for Covariates

| **Primary Measures and Covariates** | **Odds ratio (95% CI)** | | | |
| --- | --- | --- | --- | --- |
|  | **Loneliness at time 1** | **Frailty at time 1** | **Loneliness at time 2** | **Frailty at time 2** |
| **Loneliness at time 1 (vs no loneliness)** | - | 0.88 (0.45-1.71) | 13.38 (8.95-19.99) | 2.27 (1.25-4.11) |
| **Loneliness at time 2 (vs no loneliness)** | - | - | - | - |
| **Frailty at time 1 (vs no frailty)** | 0.88 (0.45-1.72) | - | 0.95 (0.39-2.29) | 28.63 (13.44-61.01) |
| **Frailty at time 2 (vs no frailty)** | - | - | 1.47 (0.74-2.92) | - |
| **≥60 y (vs <60 y)** | 1.68 (1.15-2.45) | 5.74 (2.58-12.76) | 0.80 (0.52-1.23) | 2.38 (1.24-4.58) |
| **Race and ethnicity** |  |  |  |  |
| Black (vs White) | 1.07 (0.68-1.69) | 0.98 (0.43-2.27) | 1.38 (0.82-2.31) | 2.52 (1.26-5.05) |
| Hispanic (vs White) | 0.74 (0.39-1.41) | 1.47 (0.50-4.32) | 0.95 (0.47-1.95) | 1.49 (0.53-4.22) |
| Other (vs White) | 0.50 (0.15-1.69) | 1.97 (0.37-10.35) | 0.23 (0.05-1.12) | 0.41 (0.04-4.16) |
| **Education** |  |  |  |  |
| Less than high school (vs high school) | 1.45 (0.44-4.77) | 2.18 (0.43-11.16) | 1.94 (0.53-7.14) | 0.40 (0.07-2.41) |
| At least some college (vs high school) | 0.84 (0.46-1.52) | 0.67 (0.26-1.69) | 1.29 (0.65-2.57) | 0.51 (0.21-1.23) |
| Graduate school (vs high school) | 0.75 (0.40-1.38) | 0.40 (0.14-1.11) | 1.14 (0.56-2.31) | 0.82 (0.34-2.01) |
| **Participants living with HIV (vs Participants living without HIV)** | 1.33 (0.93-1.89) | 1.23 (0.65-2.34) | 0.68 (0.45-1.03) | 0.71 (0.39-1.27) |
| **Age discrepancy** |  |  |  |  |
| Younger (vs no age discrepancy) | 0.59 (0.33-1.06) | 0.38 (0.15-0.95) | 0.66 (0.34-1.28) | 0.81 (0.31-2.12) |
| Older (vs no age discrepancy) | 1.84 (0.79-4.29) | 2.73 (0.91-8.17) | 1.97 (0.75-5.20) | 1.93 (0.58-6.46) |
| **Depressive symptoms (vs no depressive symptoms)** | 7.01 (4.54-10.81) | 2.54 (1.29 – 5.00) | 1.74 (1.05-2.89) | 1.22 (0.62-2.43) |
| **Comorbidities** |  |  |  |  |
| Each increase in count of comorbidities | 1.11 (0.94-1.31) | 1.38 (1.04-1.83) | 1.17 (0.97-1.41) | 1.34 (1.04-1.74) |
